# Choriocarcinoma Syndrome: A Case Report and a Literature Review

**DOI:** 10.1155/2013/697251

**Published:** 2013-05-29

**Authors:** Khaled Baagar, Fahmi Yousef Khan, Einas AlKuwari

**Affiliations:** ^1^Department of Medicine, Hamad General Hospital, P.O. Box 3050, Doha, Qatar; ^2^Department of Pathology and Laboratory Medicine, Hamad General Hospital, P.O. Box 3050, Doha, Qatar

## Abstract

A 35-year-old Qatari man presented to our hospital with a 4-month history of mild abdominal pain, weight loss, and jaundice. He was found to have central intra-abdominal mass and a single testis in the scrotum. His investigations showed cholestatic jaundice and very high level of **β**-HCG (1131379 IU/L). CT scans of the chest and abdomen showed a huge pelvic-abdominal mass with extensive retroperitoneal lymphadenopathy, in addition to liver and lung metastases. CT-guided Tru-Cut biopsy of the mass showed mixed germ cell tumor. Chemotherapy was refused by the patient and his family. In the following days, the patient bled from his liver metastases leading to hemorrhagic shock, hemorrhage from metastatic sites of choriocarcinoma containing tumors is named choriocarcinoma syndrome. He was transferred to the medical intensive care unit, where he was intubated and resuscitated. Embolization of the right hepatic artery was done, but failed to control the bleeding, which continued with development of disseminated intravascular coagulopathy and a severe abdominal compartment syndrome, and eventually the patient died.

## 1. Introduction

Testicular neoplasms comprise the most common solid malignancy affecting men between the ages of 15 and 35, but they only represent almost 1% of all solid tumors in males. Approximately two to three new cases per 100,000 males are reported in the United States each year, and 95 percent of all primary testicular tumors are germ cell tumors [1, page 1574]. This rare tumor can be complicated by a very rare, life threatening complication which is choriocarcinoma syndrome with only few cases reported worldwide. Choriocarcinoma syndrome entails hemorrhage from metastatic sites of choriocarcinoma associated with a significant rise of beta-human chorionic gonadotropin (*β*-HCG) [2]. In this report, we present a case of choriocarcinoma syndrome in a 35-year-old Qatari man, to draw attention of our physicians to the importance of considering this syndrome while dealing with patients who have massive and advanced testicular tumors with high *β*-HCG, as this syndrome is life-threatening and needs urgent treatment. 

## 2. Case Report

 A 35-year-old male, not known to have any chronic illness, presented with a 4-month history of mild, dull, and intermittent abdominal pain, associated with jaundice, loss of appetite, and weight loss of 10 kilograms, as well as a one-month history of abdominal swelling.

**Figure 1:**
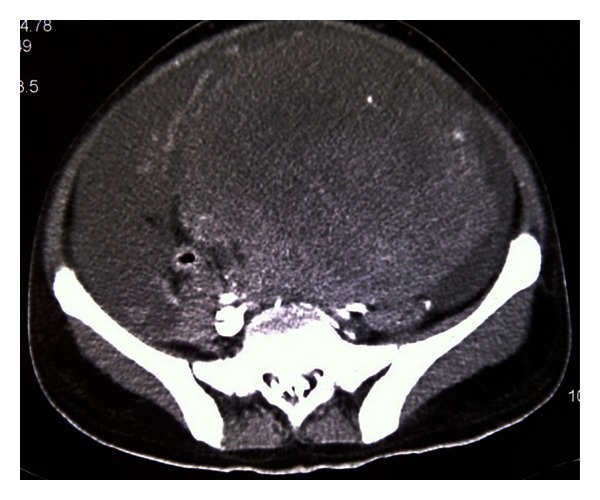
CT abdomen at presentation showed huge mass.

**Figure 2:**
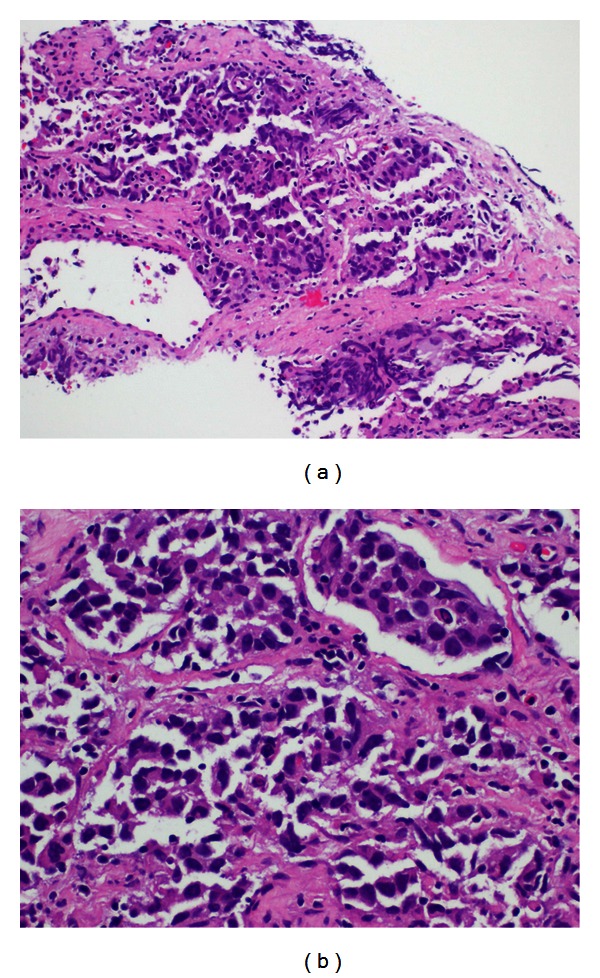
(a) Foci of neoplastic cells consistent with germ cell tumor (H&E). (b) Foci of neoplastic cells consistent with germ cell tumor (Medium Power, H&E).

**Figure 3:**
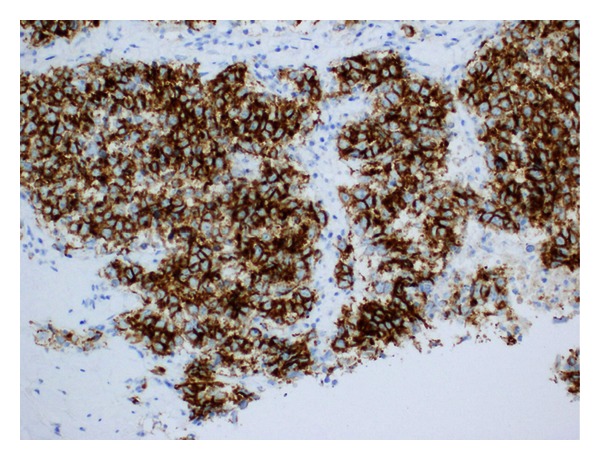
CD117 positivity in the seminomatous component.

**Figure 4:**
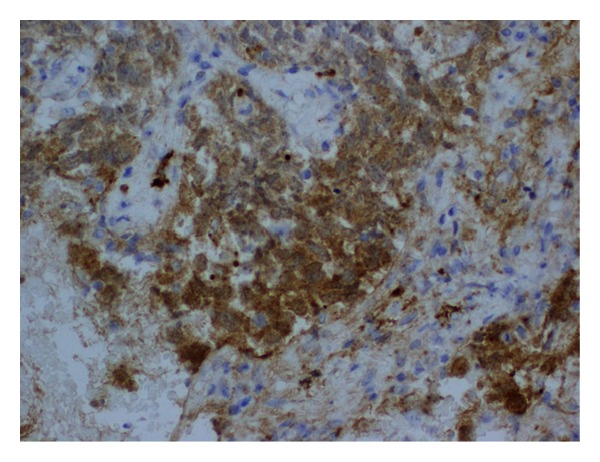
*β*-HCG positivity in foci suggestive of choriocarcinomatous component.

**Figure 5:**
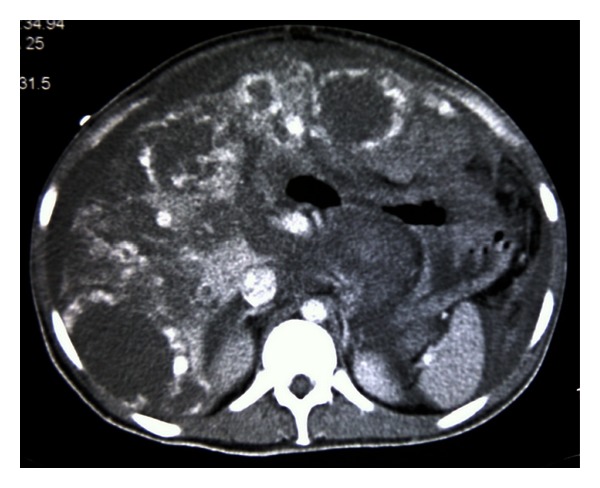
CT abdomen after development of hemorrhagic shock showed multiple heterogeneously enhancing masses in the liver with peripheral enhancement. Also, there is intraperitoneal fluid.

 On examination, he had normal vital signs, but he was pale and jaundiced. There were no palpable lymph nodes. There was central intra-abdominal mass 15 cm in diameter, firm, and tender with  smooth surface. He had single right testicle in the scrotum. Laboratory investigations (Table 1) showed white blood cells: 14.7 × 1000/mm^3^ (normal: 4 × 1000–10 × 1000/mm^3^), hemoglobin: 7.4 g/dL (normal: 13–17 g/dL), and platelets: 480 × 1000/mm^3^ (normal: 150 × 1000–400 × 1000/mm^3^). Blood chemistry and renal function were within normal limits. Aspartate aminotransferase: 53 u/L (normal: 12–39 u/L), alanine aminotransferase: 16 u/L (normal: 0–40 u/L), alkaline phosphatase: 472 u/L (normal: 40–129 u/L), gamma glutamyl transpeptidase: 155 U/L (normal: 11–50 U/L), bilirubin: 115 umol/L then 330 umol/L (normal: 3.5–24 umol/L), and direct bilirubin: 306 umol/L (normal: up to 7 umol/L). Tumor markers: *β*-HCG: 1131379 IU/L (normal: 0–5 IU/L), LDH: 2331 U/L (normal: 240–480 U/L), AFP: 1.4 IU/mL (normal: 0–5 IU/mL), CA19-9: 9 U/mL (normal: 0–37 U/mL) CEA: 1.2 Ug/L (normal: 0–3 Ug/L). FT4: 49.4 pmol/L (normal up to 20 pmol/L) and TSH: 0.01 *μ*/L (normal 0.45–4.5 *μ*/L). CT scans of chest, abdomen, and pelvis with contrast showed huge pelvic-abdominal mass measuring 19 × 14.7 × 22 cm with extensive retroperitoneal lymphadenopathy, and liver and lung metastases (Figure 1). CT-guided Tru-Cut biopsy of the mass showed a mixed germ cell tumor (Figures 2(a) and 2(b)) with predominantly seminomatous component (CD117 positive, Figure 3) and foci consistent with choriocarcinoma (*β*-HCG positive, Figure 4). Despite a high risk of intratumor bleeding attributed to tumor size and the oncologist's recommendation for immediate chemotherapy, the patient and his family refused chemotherapy as they planned to travel abroad for a second opinion. On the following days, his condition deteriorated and his level of consciousness decreased; his BP was 70/40 mmHg, pulse rate 140/min, respiratory rate 35/min, and oxygen saturation 91% with nonrebreathing mask on 15 liters oxygen/min. So, he was transferred immediately to the medical intensive care unit (MICU) where he was intubated. Laboratory investigations (Table 1) showed white blood cells: 8.4 × 1000/mm^3^, hemoglobin: 2.5 g/dL, and platelets: 113 × 1000/mm^3^. Bun: 11.5 mmol/L (normal: 1.7–8.3 mmol/L), creatinine: 222 umol/L (normal: 62–124 umol/L), K: 5.2 mmol/L (normal: 3.6–5.1 mmol/L), Na: 134 mmol/L (normal: 135–145 mmol/L), bicarbonate: 9 mmol/L (normal: 24–30 mmol/L), Cl: 98 mmol/L (normal: 96–110 mmol/L), bilirubin: 722 umol/L, and lactate: 5 mmol/L (normal: 0.5–2.2 mmol/L). INR: 1.4, APTT: 31 seconds (normal: 26–38.5 seconds). ABG: PH: 7.128, PO_2_: 270, PCO_2_: 22, HCO_3_: 8, and oxygen saturation: 99%. The patient was given intravenous fluids, packed red blood cells, fresh frozen plasma and cryopricepitate transfusions, and vasopressors. CT angiogram showed multiple enhancing hepatic metastasis with extensive blood leaking and pooling in the liver and to a lesser extent in the pelvic mass with large amount of intraperitoneal fluid (most likely hemoperitoneum) (Figure 5). So, angiography and embolization of the right Hepatic artery with gelfoam were done. 

 Despite these interventions, the bleeding continued. Hemoglobin and blood pressure continued dropping, and hepatic surgeon advised for conservative treatment as the patient was at high risk for bleeding (INR 2.3 and aPTT 49.20 seconds). Moreover, the patient developed DIC, evidenced by prolonged INR, aPTT, and low platelets, and activated factor seven was given.

 The patient developed anuria as his intra-abdominal pressure reached 50–55 mmHg. Surgery team was consulted for decompression laparotomy, but they thought it was useless and would not help the patient. At the end, the patient died secondary to hemorrhagic shock, DIC, and abdominal compartment syndrome with renal shutdown. 

## 3. Discussion

We present the clinical course of a patient with a metastatic testicular cancer, which developed in an undescended testicle and was complicated with choriocarcinoma syndrome as he got massive bleeding in the hepatic metastases leading to his death. To the best of our knowledge, the presence of cholestatic jaundice upon presentation, with high direct bilirubin, alkaline phosphatase, and gamma glutamyl transpeptidase and normal ALT, was only reported once as an initial symptom secondary to metastasis to hepatic hilar lymph nodes which is very rare as usually nonseminomatous germ cell tumors of the testis metastasize to the retroperitoneal lymph nodes, lung, liver, and brain [3]. Careful examination with discovery of only single testicle in the scrotum helped us to suspect the disease process behind the patient's condition, as the ultimate incidence of testicular tumors is about 0.002% in normal males and up to 5% in the case of intra-abdominal testes [1, page 1127]. 

The pathogenesis of choriocarcinoma syndrome is unknown. It may be related to tumor invasion of the small blood vessels [4].

It occurs in two different clinical settings, either few hours after initiation of combined chemotherapy which is more common or much less likely spontaneously in advanced disease without relation to treatment as in our patient [5].

Acute hemorrhage in the pulmonary metastasis is the typical presentation of choriocarcinoma syndrome; however, hemorrhage at any site of metastasis can develop [2]. In our patient, hemorrhage was from the liver metastases and this might be to some extent related to the smaller size of the lung metastases (the biggest lung nodule measured 2 × 2.5 cm, but the biggest in the liver measured 7 × 6.3 cm). 

Our patient was found to have severe hyperthyroidism. In general, hyperthyroidism rarely develops as a paraneoplastic syndrome. It presents in 3.5% of the patients with disseminated nonseminomatous germ cell tumors and 50% of the patients with HCG above 50000 IU/L versus 0% of the patients with HCG below this level [6]. Thyroid function should be checked in patients with high HCG, as hyperthyroidism symptoms resemble those of advanced metastatic disease and it can be missed. The mechanism of the hyperthyroidism is probably due to the ability of HCG to stimulate the TSH receptors, as it has an identical alpha subunit to that of the TSH [6]. Choriocarcinoma syndrome has a poor prognosis, particularly in patients with *β*-HCG level above 50000 IU/L [4]. 

This syndrome is a big challenge, where patients with disseminated disease treated with primary chemotherapy followed by surgery have a 5-year disease-free survival rate up to 80% [1, page 1575]. The usual chemotherapy protocol is the BEP therapy (bleomycin, etoposide, and cisplatinum). Choriocarcinoma syndrome needs early recognition and urgent treatment which is usually multimodal consisting of medical treatment, usually in the intensive care unit, to stabilize the patient and surgical intervention when appropriate [7].

In conclusion, this case draws attention of our physicians to the importance of considering this syndrome while dealing with patients who have massive and advanced testicular tumors with high *β*-HCG, as this syndrome is life-threatening and needs urgent treatment. 

## Figures and Tables

**Table 1:** Investigations done during the patient hospitalization.

Component	Upon presentation	In MICU	Reference range
WBC	14.7 × 1000/mm^3^	8.4 × 1000/mm^3^	4 × 1000–10 × 1000/mm^3^
Hemoglobin	7.4	2.5	13–17 g/dL
Platelets	480 × 1000/mm^3^	113 × 1000/mm^3^, then 47 × 1000/mm^3^	150 × 1000–400 × 1000/mm^3^
Creatinine	118	222	62–124 umol/L
BUN	8.3	11.5	1.7–8.3 mmol/L
Potassium	4.6	5.2	3.6–5.1 mmol/L
Sodium	134	134	135–145 mmol/L
Chloride	98	98	96–110 mmol/L
Bicarbonate	22	9	24–30 mmol/L
AST	53	4425	12–39 u/L
ALT	16	670	0–40 u/L
ALP	472	1396	40–129 u/L
GGT	155		11–50 U/L
Bilirubin	115 then 330	722	3.5–24 umol/L
Direct bilirubin	306		Up to 7 umol/L
INR	1.1	1.4 then 2.3	
APTT	28	31 then 49.20	26–38.5 seconds
*β*-HCG	1131379		0–5 IU/L
LDH	2331		240–480 U/L
AFP	1.4		0–5 IU/mL
CA19-9	9		0–37 U/mL
CEA	1.2		0–3 Ug/L
FT4	49.4		Up to 20 pmol/L
TSH	0.01		0.45–4.5 *μ*/L
Lactic acid		5, then 14.41	0.5–2.2 mmol/L

WBC: white blood cells, BUN: blood urea nitrogen, AST: aspartate aminotransferase, ALT: alanine aminotransferase, ALP: alkaline phophatase, GGT: gamma glutamyl transpeptidase, INR: international normalized ratio, APTT: activated partial thromboplastin time, *β*-HCG: *β*-human chorionic gonadotropin, LDH: lactate dehydrogenase, AFP: alpha-fetoprotein, CA19-9: carbohydrate antigen 19-9, CEA: carcinoembryonic antigen, FT4: free thyroxine, and TSH: thyroid stimulating hormone.
